# Draft Genome Sequence of *Bacillus* sp. strain YC2, an Isolate from the South African Medicinal Plant Pelargonium sidoides

**DOI:** 10.1128/mra.00879-21

**Published:** 2022-03-28

**Authors:** Aza Z. Mqulwa, Wai Yin Chan, Lauren A. du Toit, Kim M. Trollope, Heinrich Volschenk

**Affiliations:** a Department of Microbiology, Stellenbosch University, Stellenbosch, South Africa; b Department of Biochemistry, Genetics and Microbiology (BGM) and Forestry and Agricultural Biotechnology Institute (FABI), University of Pretoria, Pretoria, South Africa; c National Institute for Communicable Disease, National Health Laboratory Service, Johannesburg, South Africa; SIPBS, University of Strathclyde

## Abstract

A rhizosphere-associated *Bacillus* species was isolated from Pelargonium sidoides DC (*Geraniaceae*) tubers, whose commercial extracts are used in respiratory tract infection treatment. Genomic data for *Bacillus* isolates associated with *Pelargonium sidoides* is lacking. Here, we report the draft genome sequence of *Bacillus* sp. strain YC2.

## ANNOUNCEMENT

Pelargonium sidoides tuber extracts are used globally as commercial herbal medicines for respiratory infections (1). *In vitro* evidence detailed the antifungal activity of P. sidoides extracts against Cryptococcus neoformans (2), whose route of infection is via the inhalation of infectious particles (3, 4). Rhizospheric and endophytic microbes produce antimicrobial compounds and supply them to plant hosts or the surrounding areas. Plant growth-promoting *Bacillus* spp. are widely reported for their beneficial role in agriculture and their abundance in soil, plant rhizospheres, and within plant hosts (5). To expand the genomic information of rhizospheric and endophytic microbes, genome sequencing was performed on the *Bacillus* sp. strain YC2 associated with P. sidoides tubers.

Strain YC2 was isolated from serially diluted macerated suspensions of surface-sterilized, wild P. sidoides tubers (supplied by Parceval Pharmaceuticals, Wellington, South Africa) plated on lysogeny broth (LB) agar with 10 μg/mL nalidixic acid and cycloheximide. The pure isolate was cultured from a single-colony 5-mL preculture in 50 mL LB at 37°C and 120 rpm. After 24 h, cells were harvested (4,863 × *g*, 20 min, 25°C), and genomic DNA was extracted (6). Briefly, cells were washed with 10 mL lysis buffer (0.5 M EDTA, 0.1 M NaCl, pH 7.5), resuspended in 4 mL lysis buffer, and treated with 10 mg lysozyme at 37°C for 10 min. Thereafter, 0.3 mL 20% (wt/vol) *N*-laurylsarcosine was added. After 5 min at 37°C, 4 mL phenol was added, followed by centrifugation at 2,330 × *g* for 20 min and 25°C. Supernatant was decanted and treated with 10 μg RNase A for 2 h at 37°C. Genomic DNA was re-extracted with chloroform, ethanol precipitated, air-dried, and dissolved in 0.05 M Tris-EDTA buffer, pH 8. Concentration was determined by Qubit DNA analysis (Invitrogen, USA).

Genome sequencing was conducted by the Microbial Genome Sequencing Center in Pittsburgh, USA (https://www.migscenter.com/). Multiplexed Illumina sequencing libraries were constructed (7) and sequenced on the NextSeq 550 platform with high-output flowcell using the recommended Illumina manufacturer protocol. A total of 5,865,370 paired-end reads with a read length of 149 bp were generated. *De novo* assembly was conducted using SPAdes v3.12.0 (8) with the read coverage cutoff value of 80× after the raw sequence data were trimmed by Trim Galore v0.6.6 (9, 10) (access date 9 February 2021). Average nucleotide identity based on BLAST (ANIb) with alignment fraction (AF%) was calculated using pyani v0.2.11 (11). The assembly was annotated by NCBI Prokaryotics Genome Annotation Pipeline v5.2 (12). Default parameters were used for all software unless otherwise noted.

The draft genome of strain YC2 is 3,912,576 bp in size, with 44 contigs, *N*_50_ value of 275,976 bp, and a GC content of 45.23%. The assembly has a coverage of 334× and carries 3,876 protein coding sequences and 54 tRNA, 5 noncoding RNA (ncRNA), and 3 rRNA genes. The 16S rRNA sequence shares an 100% identity to Bacillus amyloliquefaciens strain AD2. The genome sequence shows ANIb values of 86.46 (82.5%) and 94.5 (84.4%) with Bacillus amyloliquefaciens ATCC 23350^T^ and Bacillus nakamurai CCUG68786^T^, respectively. It contains 16 putative biosynthetic gene clusters for secondary metabolites as predicted by the antiSMASH v6.0.1 database (13) (access date, 31 August 2021).

### Data availability.

The draft genome assembly for *Bacillus* sp. YC2 has been deposited at the National Center for Biotechnology Information (NCBI) with GenBank accession number JAHXDO000000000. The SRA, BioProject, and BioSample accession numbers are SRR15685468, PRJNA748011, and SAMN20305144, respectively.
